# Assistance Device Based on SSVEP-BCI Online to Control a 6-DOF Robotic Arm

**DOI:** 10.3390/s24061922

**Published:** 2024-03-17

**Authors:** Maritza Albán-Escobar, Pablo Navarrete-Arroyo, Danni Rodrigo De la Cruz-Guevara, Johanna Tobar-Quevedo

**Affiliations:** 1 Department of Energy and Mechanics Sciences, Universidad de las Fuerzas Armadas, Sangolqui 171103, Ecuador; mealban10@espe.edu.ec (M.A.-E.); jbtobar@espe.edu.ec (J.T.-Q.); 2 Department of Electrical, Electronics and Telecommunications Engineering, Universidad de las Fuerzas Armadas, Sangolqui 171103, Ecuador; 3 Department of Mechanical Engineering, Escuela Politécnica Nacional, Quito 170525, Ecuador

**Keywords:** assistance device, rehabilitation engineering, SSVEP-BCI, 6-DOF robotic arm, EEG sensors

## Abstract

This paper explores the potential benefits of integrating a brain–computer interface (BCI) utilizing the visual-evoked potential paradigm (SSVEP) with a six-degrees-of-freedom (6-DOF) robotic arm to enhance rehabilitation tools. The SSVEP-BCI employs electroencephalography (EEG) as a method of measuring neural responses inside the occipital lobe in reaction to pre-established visual stimulus frequencies. The BCI offline and online studies yielded accuracy rates of 75% and 83%, respectively, indicating the efficacy of the system in accurately detecting and capturing user intent. The robotic arm achieves planar motion by utilizing a total of five control frequencies. The results of this experiment exhibited a high level of precision and consistency, as indicated by the recorded values of ±0.85 and ±1.49 cm for accuracy and repeatability, respectively. Moreover, during the performance tests conducted with the task of constructing a square within each plane, the system demonstrated accuracy of 79% and 83%. The use of SSVEP-BCI and a robotic arm together shows promise and sets a solid foundation for the development of assistive technologies that aim to improve the health of people with amyotrophic lateral sclerosis, spina bifida, and other related diseases.

## 1. Introduction

There are a variety of disorders that can lead to substantial or complete impairment of motor function in an individual. The aforementioned constraint significantly curtails an individual’s capacity to independently engage in routine tasks and diminishes their overall standard of living. Traditional rehabilitation interventions aim to improve the patient’s condition but their effectiveness is often limited. Moreover, achieving success in these interventions requires a substantial commitment of time and effort. Numerous studies have demonstrated that the use of efficacious therapeutic methodologies can yield noteworthy enhancements in the patient’s state, notably in select instances of disorders of the nervous system [1,2,3].

The application of robotic technology in medical therapy has yielded positive results by enabling the delivery of rigorous and repetitive training. There exists a considerable body of evidence that highlights the crucial significance of robotic technology in enhancing the autonomy of individuals with disabilities. However, a notable obstacle persists for individuals who face severe impairments in their upper extremities. The practical application of technology such as wheelchairs, exoskeletons, or robotic arms can be limited due to complications in control methods which can affect their efficacy and reliability. It is imperative to confront and overcome these obstacles to fully harness the capabilities of assistive robotic technologies [4,5,6].

BCI-assisted devices have immense potential for application in a wide range of diseases, including multiple sclerosis, stroke, amyotrophic lateral sclerosis, spina bifida, muscular dystrophy, trauma, and various neurological disorders. Because it is possible to create a direct neural interface between the brain and assistive technologies, communication between muscles or peripheral nerves is not needed. The inherent nature of these technologies, together with their wide range of potential uses, renders them highly promising for offering support in diverse healthcare contexts. There are two primary ways to record brain signals from the central nervous system: invasive and noninvasive techniques [7,8].

Facilitating the acquisition of targeted brain signals is achieved through the utilization of noninvasive EEG electrodes, typically incorporated into helmets adhering to international positioning standards for precise placement on the scalp. Within the realm of BCIs, the SSVEP paradigm is frequently employed to decipher user intention. In this paradigm, observers are presented with luminous entities, geometric shapes, or visual representations that flicker at known frequencies. User intention is discerned as the observer fixates on a stimulus for a brief duration, indicating the desired course of action. The effective use of such a system hinges on the patient maintaining a functional cognitive capacity to concentrate, comprehend the system’s operation, and direct their attention accordingly. In instances where these cognitive abilities are compromised, alternative approaches, such as motor imagination coupled with additional training, warrant consideration for ensuring successful interaction with a BCI system [9,10,11,12].

The SSVEP paradigm is characterized by its minimal training requirements, making it a highly viable choice for patients engaged in rehabilitation protocols and assistance in daily tasks. Furthermore, when compared to other paradigms such as motor imagining and P300 potentials, better accuracy and information transfer rate (ITR) are achieved. The system’s low signal-to-noise ratio has facilitated its application in individuals with profound communication impairments, yielding favorable outcomes in approximately one-sixth of patients [13,14].

Previous studies have been carried out in the domain of assistive devices employing BCI-SSVEP. A brief comparison with related research is included in Table 1. The study conducted by the authors in [1] employed a BCI utilizing SSVEP to facilitate the activation of a soft robotic glove to rehabilitate patients who had experienced cerebrovascular accidents. The results of the study indicated an average accuracy of 65.89% in the BCI, based on the analysis of 10 subjects. The study conducted by the authors in reference [3] employs a similar paradigm to regulate a basic exoskeleton designed for arm rehabilitation. The system operates using three distinct control frequencies, namely, 12 Hz, 15 Hz, and 20 Hz. When conducting testing with six healthy people, an average accuracy of 80% was achieved. In a previous study conducted by the authors [15], a spelling system was developed that utilized six distinct frequencies in conjunction with SSVEP on a 24-inch display. The system underwent enhancements through the utilization of the Eyelink 1000, a highly regarded and precise eye-tracking apparatus. The authors of [16] have developed and implemented a novel approach for enhancing the signal-to-noise ratio in SSVEP by utilizing music. The article [17] analyzes and compares the utilization of nonlinear canonical correlation analysis (NLCCA) with the CCA and LASSO techniques. The data indicate that NLCCA exhibits enhanced accuracy mainly during the first two seconds of stimulation. Conversely, LASSO exhibits diminished accuracy in all assessed situations.

The significance of this research is apparent through the integration of a robotic arm as an assistive tool with a BCI. This support system is designed considering the distinct requirements and preferences of the intended user and guaranteeing the inclusion of several essential functions. The BCI achieves an accuracy of 75% during the online session and an accuracy of 83% during the offline session. This is accomplished using five control frequencies (12.25, 13.25, 14, 15.25, and 16.25 Hz). The performance factors for the BCI are determined by carrying out experimental tests on users with different characteristics. The usefulness of the BCI is further evaluated through surveys of participating users to assess the level of comfort and ease of use and to identify areas for improvement. The processing exhibits lower computational complexity by relying on mathematical relationships. This enables rapid processing, a reduced error rate, and operation without previous algorithm training. The 6-DOF robotic arm allows for accurate manipulation in three-dimensional space, providing control over both position and orientation at low speeds, ensuring stable movements, and minimizing vibrations. Finally, the implementation of plane-constraint allows the user to interact more easily with this technology, facilitating the subsequent integration of additional trajectories and movements that allow daily activities such as feeding and brushing teeth, among others.

## 2. Materials and Methods

This section explains the conceptual basis for the algorithms used, the device requirements, and the development of the assistive device.

### 2.1. Conceptual Basis

Presented below is a collection of concepts and their corresponding mathematical underpinnings that are relevant to the development of the BCI.

#### 2.1.1. Principal Component Analysis

Principal component analysis (PCA) is a technique employed to eliminate the correlation among output variables by considering their variances. This approach is based on the premise that signals exhibiting higher variances typically include relevant information, while those with lower variances represent noise or redundant data that can be disregarded [19,20].

#### 2.1.2. Independent Component Analysis

Independent component analysis (ICA) is a linear decomposition technique used to extract independent signals from a given signal. Its primary objective is to separate mixed signals within an EEG signal, grouping them based on data similarity. This allows for the isolation of signals that are of interest while disregarding unwanted signals, such as artifacts [21,22,23].

#### 2.1.3. Fast Fourier Transform

The fast Fourier transform (FFT) is a computational algorithm that efficiently computes the discrete Fourier transform. The Fourier transform can be obtained by discretizing the function f(t) into *N* values $f\left(t_{1}\right),\ldots ,f\left(t_{N}\right)$. In this case, the Fourier transform can be expressed as the summation given by Equation (1), where *k* represents the discrete frequency [24].

(1) $$F\left(n\right)=\sum_{k=1}^{N}f\left(t_{k}\right)e^{\frac{-j2\pi n}{N}\left(k-1\right)},1\leq n\leq N$$

#### 2.1.4. Power Spectral Density

The power spectral density (PSD) is a mathematical function used to quantify the power or intensity of the frequency components in a signal, measured in magnitude squared per hertz. It enables the identification of resonances and concealed harmonics in signals characterized by random vibrations, as expressed by the summation Equation (2) [25,26].

(2) P(fk)=1N|∑n=0Nx(n)e−j2πfk|2

#### 2.1.5. Canonical Correlation Analysis

Canonical correlation analysis (CCA) is a multivariate statistical method used to examine the relationship between two signals by measuring their correlation percentage, which serves as an indicator of their similarity. In the context of the SSVEP paradigm, it is necessary to conduct a comprehensive comparison between the analyzed signal and all the frequencies of the stimuli to ascertain the frequency that exhibits the highest correlation index [27,28]. Moreover, Equation (3) encompasses the consideration of several harmonics.

(3) $$Y=\left[\begin{array}{c} \sin\left(2\pi f_{k}t\right)\\ \cos\left(2\pi f_{k}t\right)\\ \vdots \\ \sin\left(2\pi N_{h}f_{k}t\right)\\ \cos\left(2\pi N_{h}f_{k}t\right) \end{array}\right]$$

Given the linear combinations of variables *X* and *Y* as depicted in Equations (4) and (5):

(4) $$x=X^{T}W_{x}$$

(5) $$y=Y^{T}W_{y}$$

The vector *X* denotes the EEG signal for *N* channels, whereas the vector *Y* represents the reference signals. Equation (6) facilitates the computation of the overall correlation:

(6) $$\begin{array}{cc} \begin{array}{c} rc\rho \left(x,y\right)= \end{array} & \frac{E\left[x^{T}y\right]}{E\left[x^{T}x\right]E\left[y^{T}y\right]}\\ \;= & \frac{E\left[W_{x}^{T}XY^{T}W_{y}\right]}{\sqrt{E\left[W_{x}^{T}XX^{T}W_{x}\right]E\left[W_{x}^{T}YY^{T}W_{y}\right]}} \end{array}$$

### 2.2. Device Requirements

During consultations with healthcare specialists and an in-depth inspection of relevant papers, certain prerequisites were identified.

- The primary responsibility of the robot is to guarantee the preservation of the user’s physical and mental well-being.
- The whole functioning should be readily comprehensible.
- The workspace of the robotic arm must be adequate to perform the tasks it is designed to facilitate.
- The cost holds significant importance.
- The weight should be minimized to the greatest extent practicable.
- It is imperative to ensure that the functioning of the robotic arm does not impede the process of acquiring the signal from the EEG signaling helmet.
- The system should make it easy to undertake maintenance and configuration processes.

Moreover, supplementary data about this research were provided. The utilization of an assistive robotic arm offers notable advantages for those with substantial disabilities, particularly concerning their upper extremities. The presence of a nurse or family member is crucial for providing supplementary support. Feeding and personal grooming are considered to be the primary actions of utmost significance for assistive technology. The aesthetic design of a product is contingent upon the preferences and choices of the consumer. However, it is recommended to choose neutral or metallic hues for the design. Regarding the potential application of this technology as a therapeutic intervention, it has been identified that certain medical conditions necessitate not only physical muscle movement but also the engagement of mental imagery and concentration, as exemplified by thrombosis.

### 2.3. Development of the Assistance Device

This section presents a comprehensive overview of the design and operation of the assistive device. This involves the development of the robotic arm and BCI, as well as the process of combining them to create a functional assistive device.

#### 2.3.1. Robotic Arm Design

The robotic arm was designed considering important calculations to obtain the direct and inverse kinematics, using computational tools to validate important factors such as inertia and torque, among others. Finally, we present the workspace obtained.

#### Direct and Inverse Kinematics

**Figure 1:**
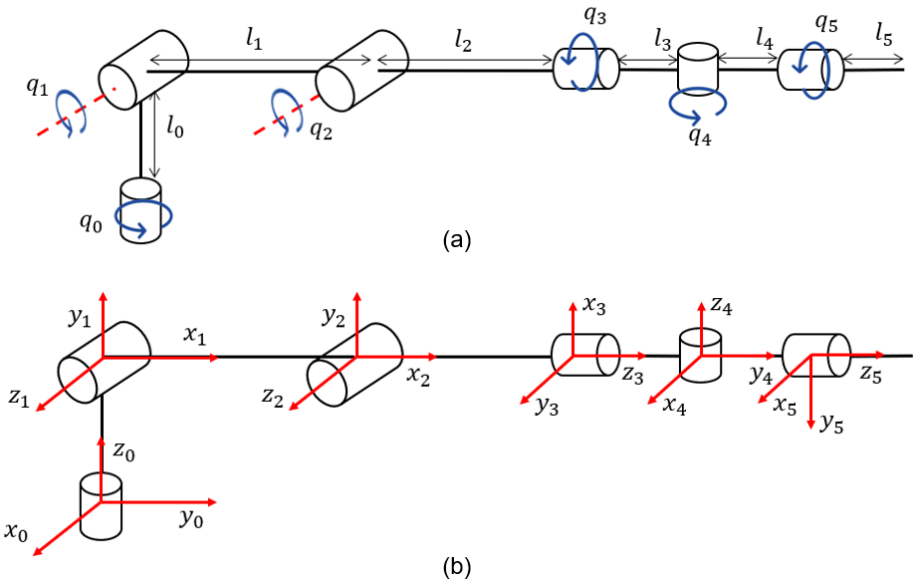
Robot arm kinematic chain: (**a**) Direction of joint movement and, (**b**) Positioning of the axes.

The use of DH parameters and kinematic decoupling methods was very important in this process; they made it easier to fully understand the arm’s shape and workspace. The utilization of three-dimensional modeling software was highly beneficial, as it facilitated a comprehensive exploration of the arm’s geometry and crucial physical attributes. The kinematic chain for the mechanical design of the robotic arm was built by utilizing a series of reference systems corresponding to each joint, as depicted in Figure 1. The direct kinematics of the robot were determined using the Denavit–Hartenberg parameters.

**Figure 2:**
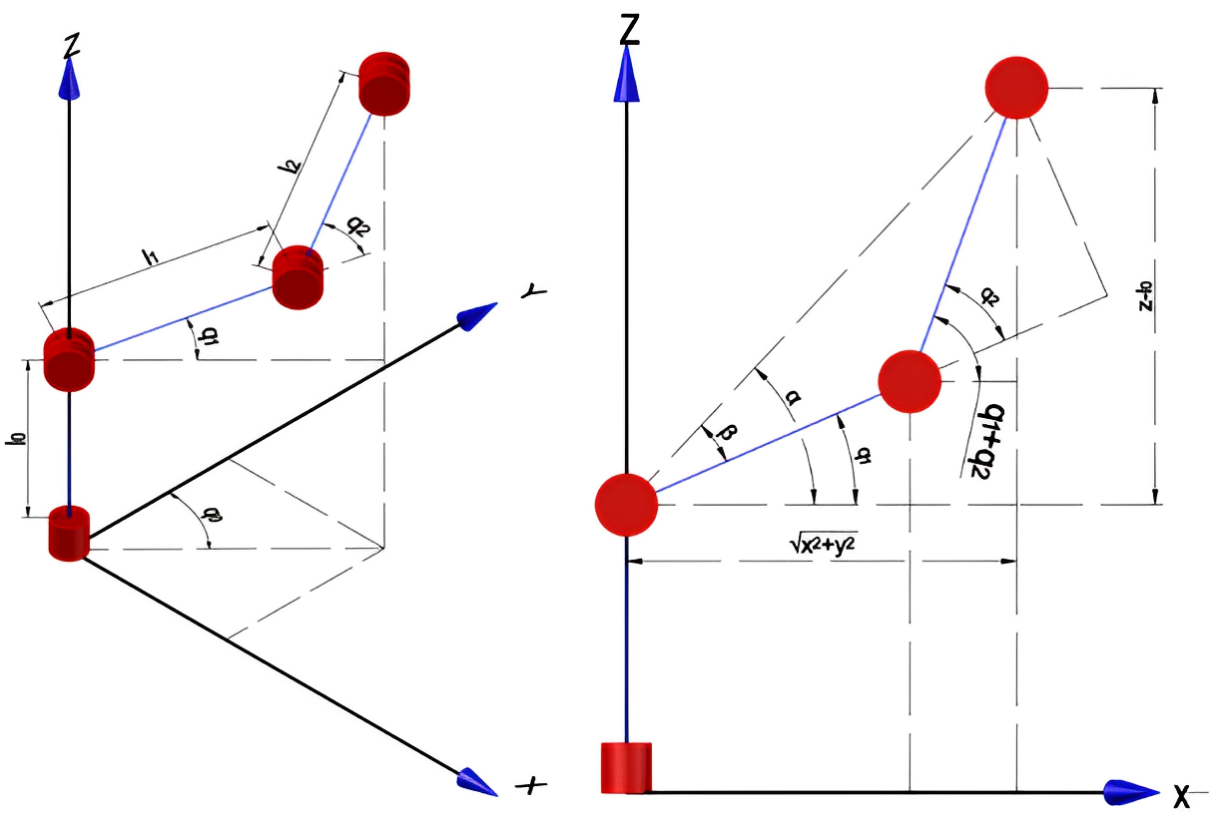
Mates of the first three links of the robotic arm.

In the context of inverse kinematics, kinematic decoupling is employed as a technique to address the problem by segregating it into distinct components about position and orientation. The utilization of the graphic method is discussed in the initial three references, as illustrated in Figure 2.

The coordinates [xc,yc,zc] represent the spatial location of the robotic wrist, as observed in Equation (7). Let $r_{13},r_{23},$ and $r_{33}$ denote the elements of the rotation matrix associated with the manipulator. In light of this, we may express Equation (8). To determine the orientation of the manipulator, it is necessary to obtain the rotation matrix O30 for the first three links. The aforementioned result was derived using direct kinematics and is represented by Equation (9). The matrix R63 is derived by the procedure outlined in Equation (10).

(7) O30=xcyczc1=x−l4r13y−l4r23z−l4r331

(8) R60=r11r12r13r21r22r23r31r32r33F

(9) $$R_{3}=\left[\begin{array}{ccc} \sin q_{0}\sin\left(q_{1}+q_{2}\right) & \cos q_{0} & -\sin q_{0}\cos\left(q_{1}+q_{2}\right)\\ -\cos q_{0}\sin\left(q_{1}+q_{2}\right) & \sin q_{0} & \cos q_{0}\cos\left(q_{1}+q_{2}\right)\\ \cos\left(q_{1}+q_{2}\right) & 0 & \sin\left(q_{1}+q_{2}\right) \end{array}\right]$$

(10) R63=R3TR6

Furthermore, the Euler angles denoted as ZXZ are regarded as observed in Equation (11) for the reference systems associated with the orientation, specifically in the final three links. The constituent elements of the R63 robot are established, thereby enabling the resolution of its inverse kinematics.

(11) R63=rotzα∗rotxγ∗rotzβ

The dynamics of the robot are influenced by various factors such as kinematics, geometry properties, masses, and the inertia of the linkages. This enables the determination of the required torque of the motors to facilitate the movement of the inertia associated with each link and the predetermined load.

By utilizing the geometric Jacobian, the linear and angular velocities can be ascertained through calculation. The Euler–Lagrange model is employed to comprehensively establish the dynamics of the manipulator based on its kinetic and potential energy.

#### Computational Design of the Robotic Arm

**Figure 3:**
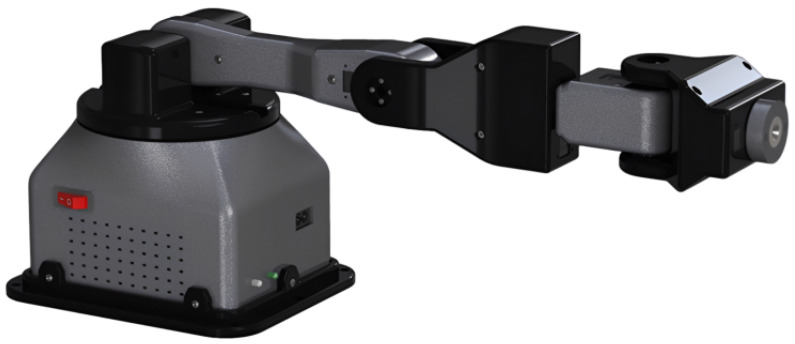
Computer-aided design of the robotic arm.

The design process is characterized by iteration, wherein one begins with a foundational geometry or maximum torque constraints and adjusts geometric parameters until the specified requirements are satisfied. The virtual design was conducted and its mechanical properties were validated, resulting in the geometry depicted in Figure 3. The aforementioned design exhibits notable attributes of stability, hardness, and mechanical resilience due to its inherent symmetry and utilization of bearings. Furthermore, the design of the robotic arm exhibits modularity and facilitates effortless assembly.

The utilization of computer-aided design facilitated the acquisition of several properties, including weights, inertia, and general geometries. These properties were then utilized to conduct a dynamic analysis, enabling the calculation of torques until the desired requirements were satisfied. The decision was made to utilize a hexagonal shape for features such as screw holes, as this configuration enables them to be discreetly concealed inside the structure while also facilitating the effective tightening of nuts.

A static analysis was conducted for every link. The experimental setup commenced with the attachment of link 6, which bore a weight of 350 g. A safety factor of 1.2 was applied to ensure structural integrity. The load point was established at each engine’s support, while the fastening sites where the propeller of the preceding engine was screwed served as additional points of support. The analysis was conducted using the most critical positions. A maximum Von Mises stress of 425 MPa in link 2 is evidence that it exhibits the highest level of criticality, according to the findings.

Three-dimensional printing was employed for the construction process owing to its advantageous ability to achieve intricate geometries with exceptional precision and visual appeal. The selection of PETG as the material for the links was based on its favorable interlayer adhesion, printing convenience, and notable resistance to impact and fatigue. Furthermore, PLA+ was employed due to its good characteristics, such as enhanced printability, superior surface quality, and notable resilience to both tension and bending. This selection was specifically made to construct the static structural components which house the electrical parts. The printing parameters were established based on the recommendations provided by the respective filament suppliers, in conjunction with conducting preliminary printing experiments.

#### Workspace of the Robotic Arm

**Figure 4:**
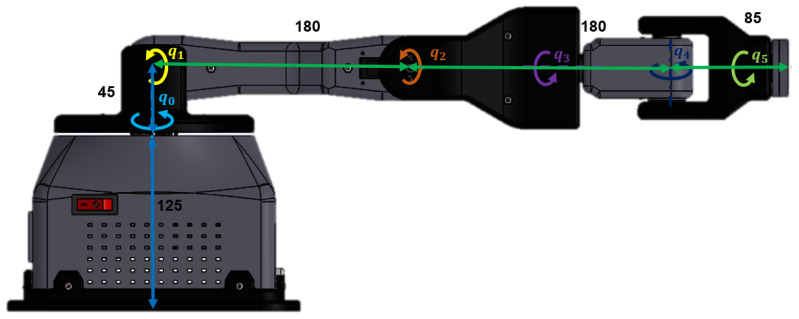
General measurements of the robotic arm.

The dimensions of the robotic arm are depicted in Figure 4. In addition, the extent of rotation for each joint aligns with the values presented in Equation (12).

(12) $$\left.\begin{array}{c} -120^{\circ }<q_{0}<120^{\circ }\\ 0<q_{1}<180^{\circ }\\ -120^{\circ }<q_{2}<120^{\circ }\\ 0^{\circ }<q_{3}<180^{\circ }\\ -90^{\circ }<q_{4}<90^{\circ }\\ 0^{\circ }<q_{5}<180^{\circ } \end{array}\right\}$$

**Figure 5:**
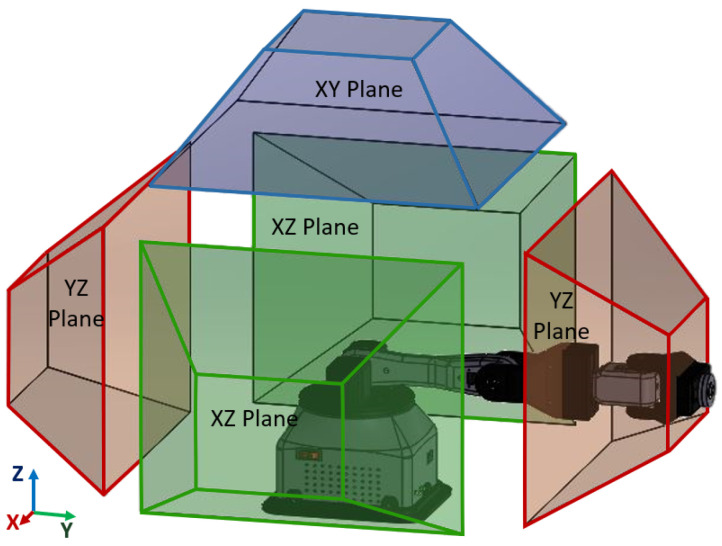
Defined workspace of the robotic arm.

The identification of the major workspace was based on the assumption of a stationary robotic wrist. The movement of the object exhibits characteristics akin to those of a hemispherical shell with a diameter measuring 85 mm. Establishing a manipulator orientation in each quadrant allowed for the conduct of graphical motion analysis. The workspace for each plane was determined and subsequently reduced to a specific area where the relevant planar zones depicted in Figure 5 could be obtained. The blue hue symbolizes the upper quadrant in the XY plane, while the green hue signifies the lateral quadrants in the XZ plane. Lastly, the red hue indicates the front and rear quadrants in the YZ plane.

#### 2.3.2. BCI Design

**Figure 6:**
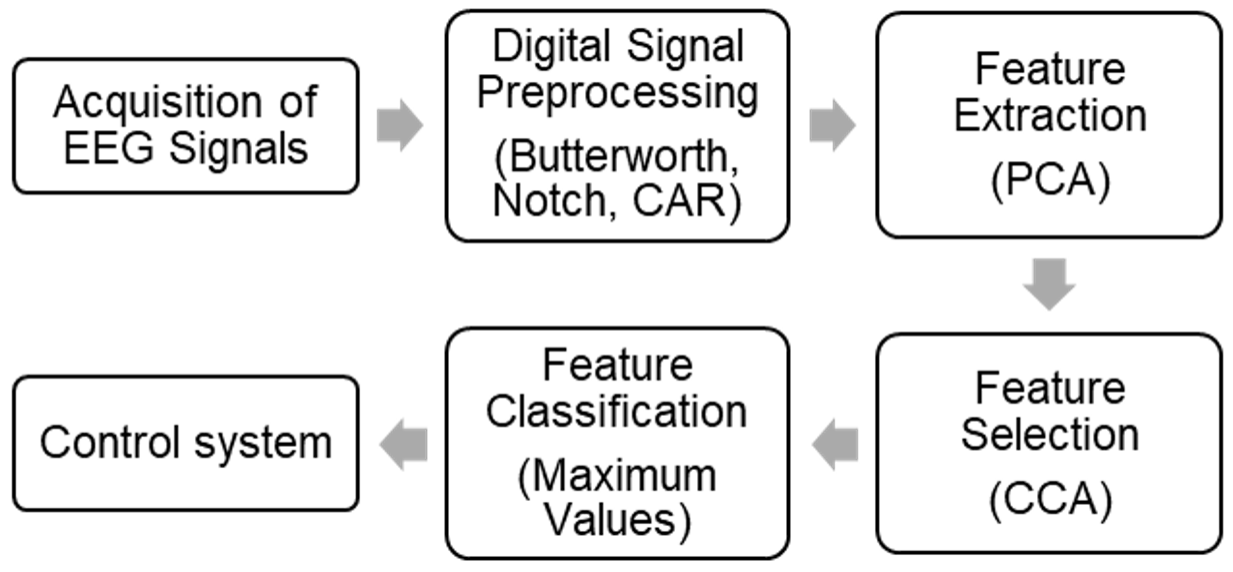
Sequential diagram of the BCI Architecture.

This section explains the design of the BCI. The acquired EEG signals go through different stages. The architecture is shown in Figure 6.

#### Signal Acquisition

**Figure 7:**
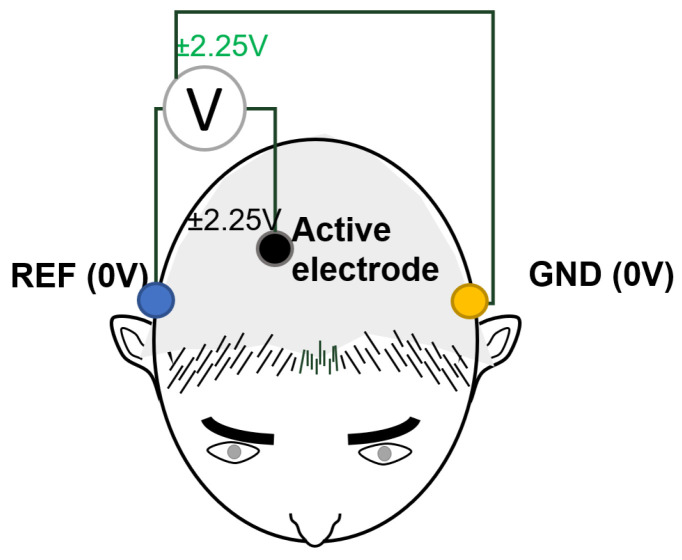
Reference scheme for EEG signal acquisition.

The gNautilus Research helmet with 64 g SAHARA electrodes from the Gtec brand was used as the receiver of the EEG signals. This features 24-bit precision at a 250 Hz sampling rate, 2.4 GHz digital transmission, and a range of 10 m. This helmet utilizes an application programming interface that has been specifically built by the manufacturer to facilitate signal acquisition. To establish a reference signal (REF) and a virtual ground (GND), the electrodes were positioned on the mastoids, as depicted in Figure 7. The resolution of the analog-to-digital converter is ±2.25 volts. The electrodes were strategically positioned in proximity to the parietal and occipital lobes because of the enhanced precision observed during the experimental procedure. The electrodes chosen for the study were P4, P6, P07, P03, P04, and POZ, which follow the internationally recognized 10–20 electrode placement system.

#### Preprocessing

During this particular stage, several filters were employed, including the Butterworth, Notch, and Common Average Reference (CAR) filters. The specific parameters of these filters may be found in Table 2.

#### Feature Extraction

An experimental methodology was employed to evaluate the performance of the PCA and ICA algorithms in decomposing the signal. The aim was to identify the technique that yielded the most favorable outcomes.

#### Feature Selection

Feature selection can be analyzed in the frequency domain using algorithms like FFT or PSD, and in the time domain using CCA. This stage was incorporated into the experimental design to determine the algorithm that achieved the highest level of accuracy [29,30].

#### Feature Classification

The activation frequency is chosen from the known stimulus frequencies based on either a higher correlation value (CCA) or a greater peak amplitude (FFT or PSD).

#### BCI Interface

**Figure 8:**
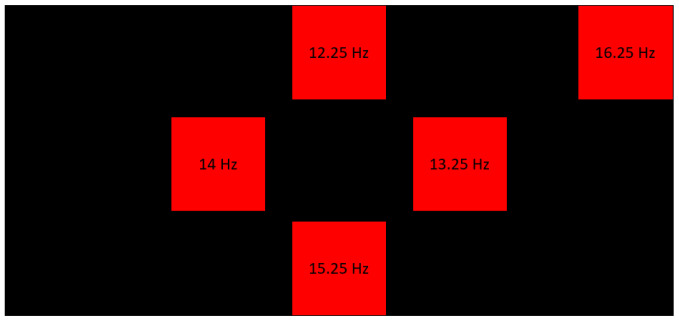
Distribution of visual stimuli on the screen of the BCI interface.

A BCI interface with a straightforward and uncluttered design was implemented to reduce potential distractions. The system’s design is structured around three distinct windows, namely, the start window, the plane selection window, and the movement selection window. The initial screen is designated for the activation of the system, whereas the subsequent two screens employ the SSVEP paradigm. Users concentrate their attention on the red boxes that correlate to the desired action for the robotic arm, specifically during the defined stimulation period. The color red was selected for its high visual appeal, enhancing the patient’s focus and leading to better outcomes. The frequencies selected for the implementation of the SSVEP paradigm are 12.25, 13.25, 14, 15.25, and 16.25 Hz, as depicted in Figure 8.

**Figure 9:**
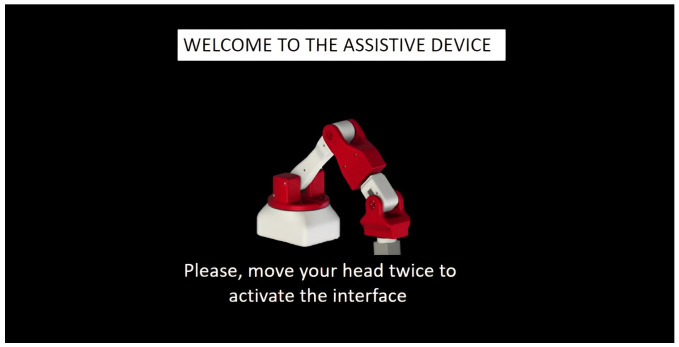
Start window of the BCI interface.

The start window presents research-related referential data along with explanations of broad indicators. In order to proceed to the subsequent window, the user is required to engage in head motions for 2 s (see Figure 9).

**Figure 10:**
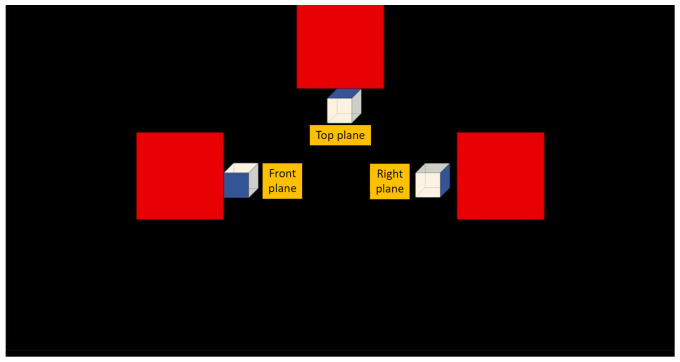
Plane selection window of the BCI interface.

The plan selection window displays three plane options: top, front, and right side (see Figure 10). The robotic arm will be directed to the initial location of the selected plane and subsequently track the designated movements.

**Figure 11:**
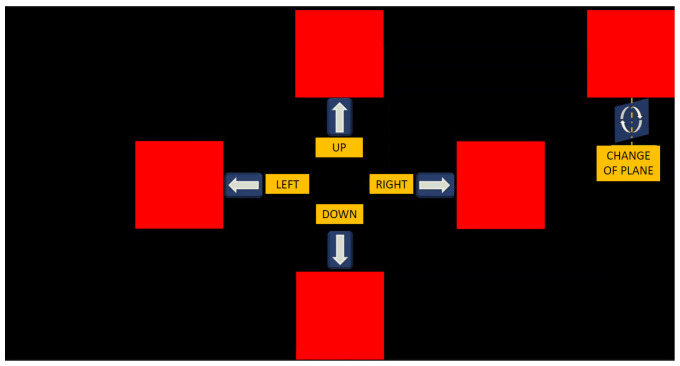
Movement selection window of the BCI interface.

The movement selection window encompasses a set of five instructions: up, down, left, right, and change of plane. These commands are associated with the movement direction of the robotic arm and facilitate the transition to a different working plane, as depicted in Figure 11.

#### 2.3.3. Assistance Device Operation

**Figure 12:**
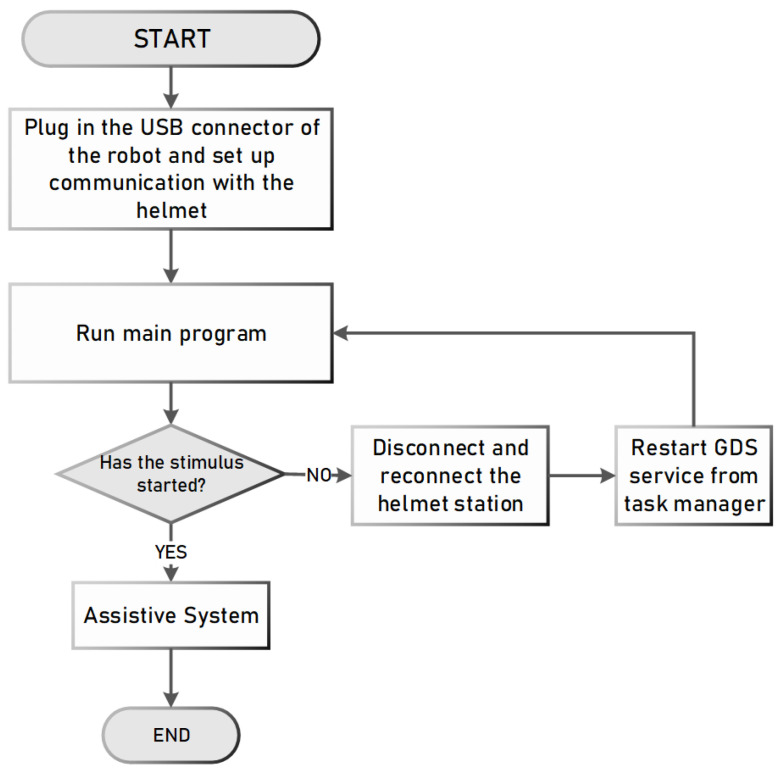
Flow diagram of the operation of the assistance system.

**Figure 13:**
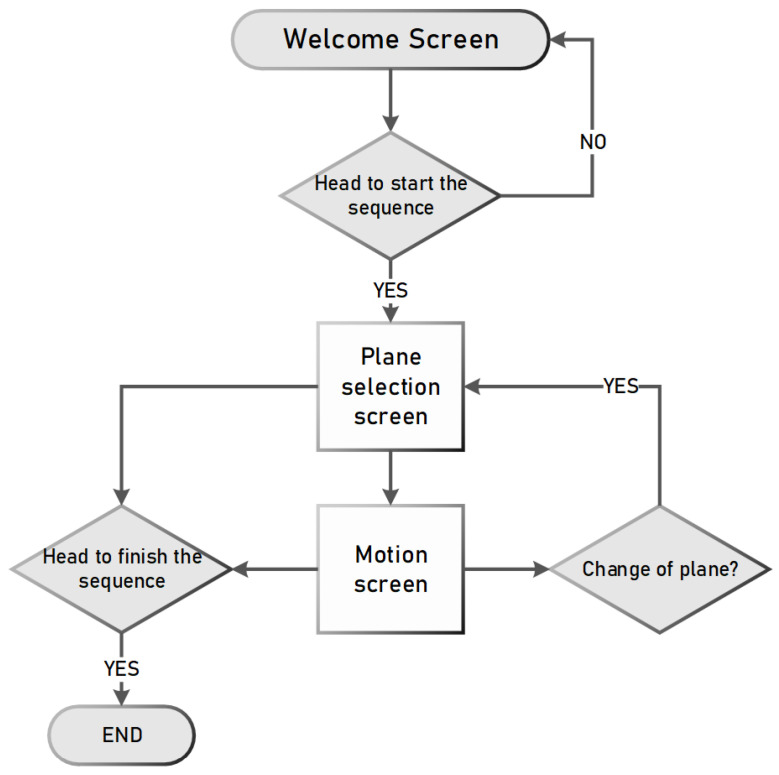
Flow diagram of the operation of the BCI.

The implementation of the assistive device was carried out in a straightforward and user-centric manner. The operational sequence is illustrated in Figure 12. It is important to establish a connection between the robotic arm and the program, ensuring that the stimulus is activated. Alternatively, the helmet facilitates the process of restarting, enabling the reestablishment of the station’s connection with the computer. Once the sequence has been launched, the user should proceed with the process as depicted in Figure 13.

Subsequently, the user is required to perform a lateral movement of their head in a reciprocating manner to initiate the sequence. After the initiation of the sequence, the plane selection window becomes visible, allowing the user to direct their attention towards the preferred plane. Subsequently, the movement is produced in the robotic arm. Depending on the chosen plane, the operator is currently able to move the robot along either the vertical or horizontal axes. An alternative option is to choose a plane alteration to revert to the preceding window. If the user shakes their head in either of the two windows, the sequence will terminate.

**Figure 14:**
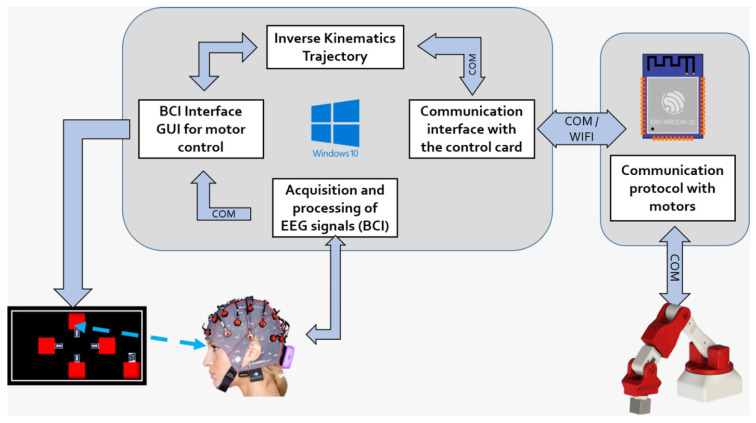
Flow diagram of the operation of the assistance device.

The signals that have been obtained are subjected to processing using the algorithms that have been discussed to discern the intention of the user. Subsequently, a directive is issued to the inverse kinematics algorithm to convert it into angular displacements of the robotic arm. The movement of the manipulator through the motors is obtained by sending these signals to a control card. The schematic representation of this process is depicted in Figure 14.

The circuit design stands out in its simplicity, as its primary function is to establish communication between the BCI and the robot’s motors while also providing them with the necessary power supply. The control card utilized in this study is an ESP32. To mitigate the risk of motor energization during instances of critically low battery levels, a relay mechanism was incorporated into the system. This feature enables the preservation of both the user’s integrity and the battery’s integrity.

The aggregate computed energy consumption corresponds to around 6.19 A. The 5000 mAh battery that is currently available has an estimated duration of around 30 min when used continuously. To account for the intermittent load-bearing nature of the arm, a low-consumption sequence has been implemented. This sequence includes the activation of a power-saving mode when 1 minute elapses without any commands being received. The utilization of this operational mode is prevalent in the context of BCIs due to the propensity of this paradigm to induce visual fatigue. Consequently, it is advisable to employ this mode for brief durations.

## 3. Experiments and Results

To assess the accuracy and ITR of the offline BCI, the primary participant (a subject with prior expertise in SSVEP) was positioned at a distance of 50 cm from the display. The researcher conducted 100 experiments, distributing them evenly between the different frequencies. Each experiment consisted of a stimulus duration of 4 s per trial and a 1 s rest period.

The online BCI was assessed using the primary participant (S00) as well as five other healthy participants (S01–S05). Initially, a comprehensive elucidation was provided regarding the overall functioning of the BCI, the requisite protocol to be executed, and the attendant hazards. The experimental procedure was conducted according to the guidelines outlined by the ethics committee, specified in the approval document 002-020.

**Figure 15:**
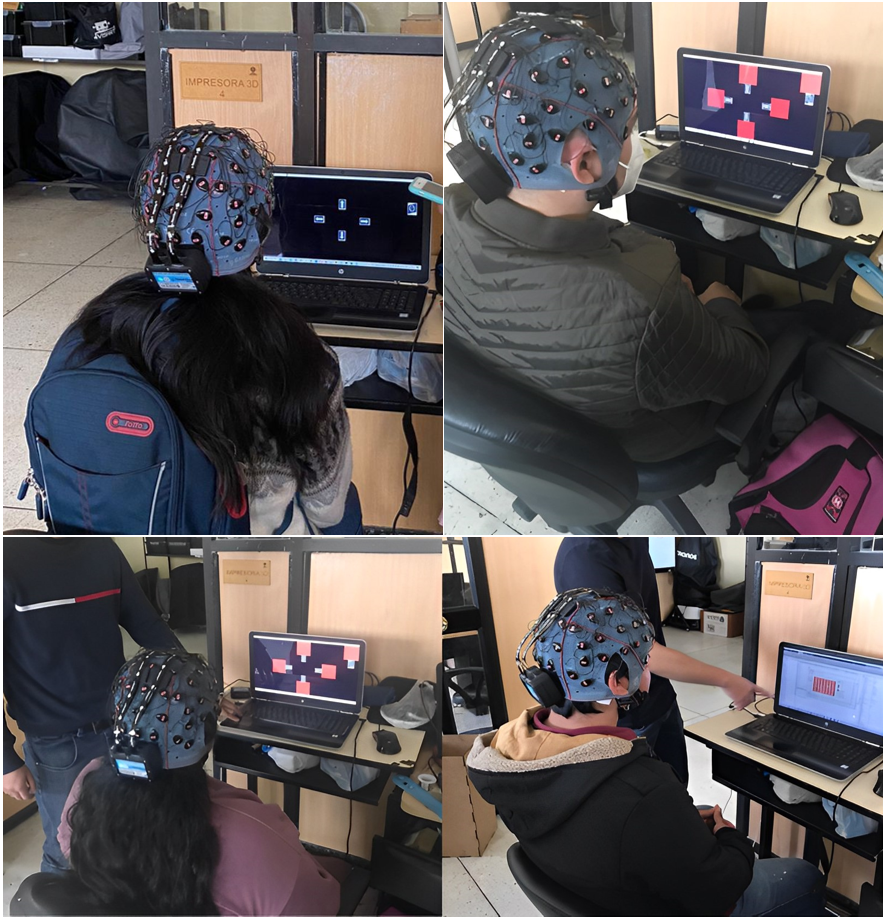
Online testing of the BCI with volunteer users with different characteristics.

The participants were positioned at a distance of 50 cm from the screen, where they were presented with a total of 10 trials for each frequency. The duration of this signal was 4 s, followed by a rest period of 6 s, implemented to mitigate potential eye strain experienced by the user. Figure 15 illustrates the methodology employed for conducting the online testing.

### 3.1. Experimental Design of the Algorithms

The experimental design chosen for this study was a Taguchi mixed-level design with two components. The objective of the design is to achieve optimal algorithm performance during the feature extraction stage, specifically using either PCA or ICA. Additionally, the design aims to optimize feature selection by CCA, FFT, or PSD, as indicated in Table 3.

**Figure 16:**
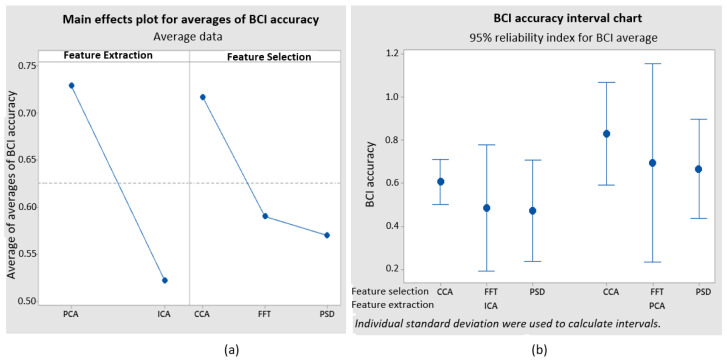
Plots resulting from the experimental design: (**a**) Main effects for averages as a function of BCI accuracy, and (**b**) BCI accuracy interval chart.

Three experimental replicates were conducted using the aforementioned algorithms. Table 4 displays the collected results. Figure 16a illustrates the major effects plot for each mean, clearly indicating that the PCA and CCA algorithms are the ones that enhance the accuracy. The analysis also assessed ITR, revealing an identical tendency. Figure 16b shows the interval graph representing the standard deviation of each set of algorithms individually. The ICA and CCA algorithms provide minimal variation, but the PCA and ICA algorithms exhibit the highest level of variation.

The methods chosen for feature extraction and feature selection were PCA and CCA, respectively. The obtained accuracy and ITR values are 83% and 20.45 bits per minute, respectively. On the other hand, the algorithms that exhibited the lowest accuracy and ITR were ICA and PSD, with respective values of 47% and 4.32 bits per minute.

### 3.2. Offline Experimental Tests of the BCI

**Figure 17:**
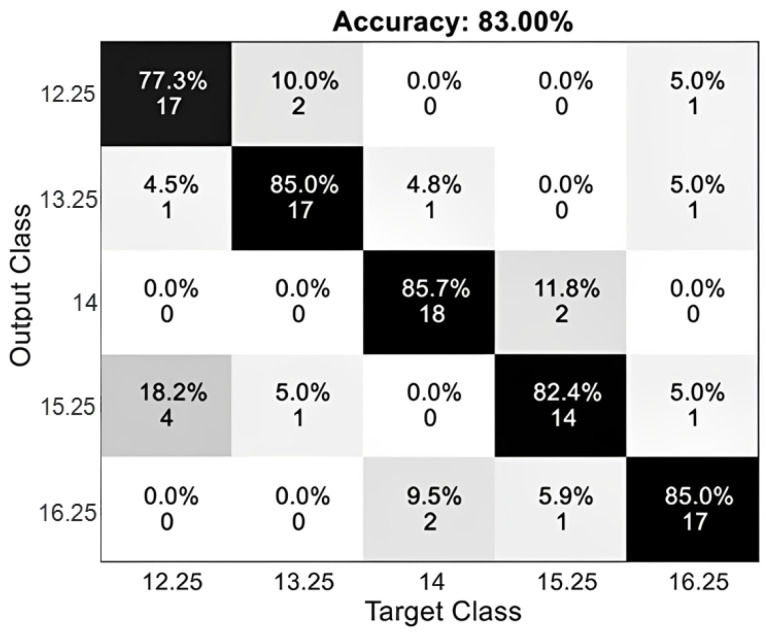
Confusion matrix offline tests.

The experimental protocol yielded the results presented in Table 5 and the confusion matrix depicted in Figure 17 during the offline tests. The confusion matrix reveals that the frequency that was most often identified was 14 Hz, with a total of 18 successful detections out of 20 sent orders. There were a total of six incorrect orders recorded at the frequency with the lowest detection rate, 15.25 Hz. Out of the aforementioned options, four of them had a frequency of 12.25 Hz, which was found to yield the highest occurrence of false negatives.

### 3.3. Online Experimental Tests of the BCI

A total of six individuals participated in the online experimental tests. The initial tests were conducted using the primary participant, S00, who possessed prior experience with SSVEP. Subsequently, more users were included in the experimentation.

#### 3.3.1. Online Tests on the Main Subject

**Figure 18:**
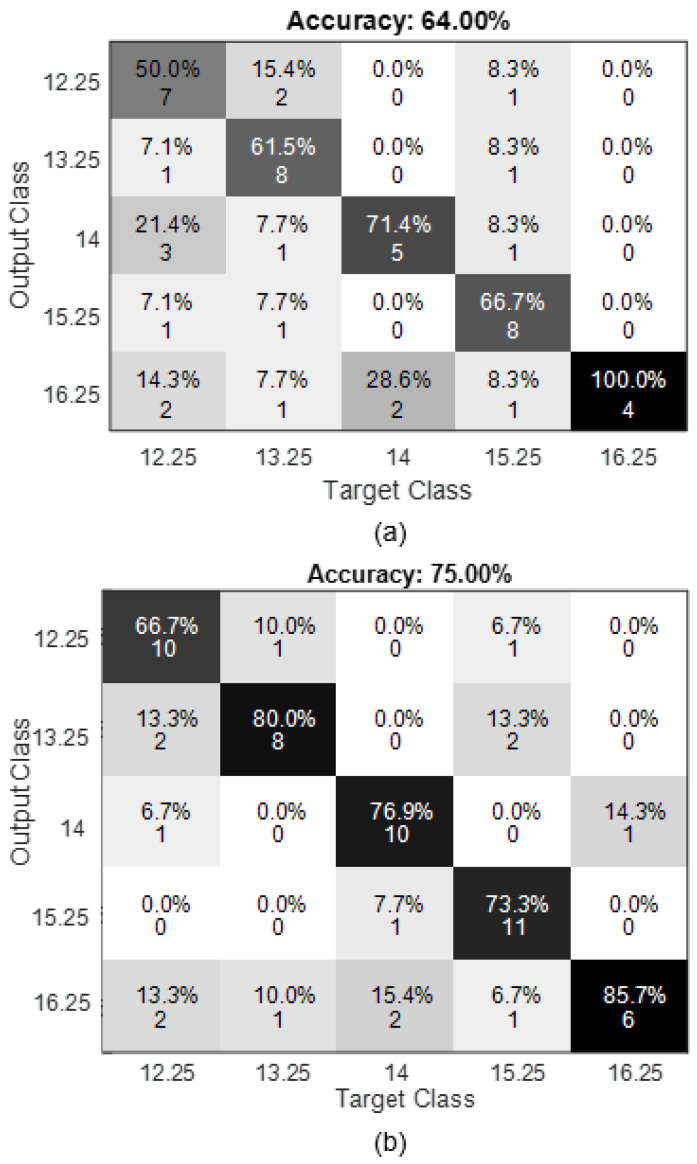
Confusion matrix online tests: (**a**) Without training, (**b**) With training.

During the initial session, when the user possessed limited familiarity with BCIs, the confusion matrix depicted in Figure 18a was acquired. Subsequently, the user was granted the opportunity to engage in practice and acclimate to the system. Ultimately, the outcomes depicted in Figure 18b were acquired. Their respective ITR and accuracies can be found in Table 6.

The improvement in accuracy was significant following the implementation of user practice with this tool. During the initial testing session, the confusion matrix revealed that the frequencies 13.25 and 15.25 Hz exhibited the highest level of detection accuracy, correctly identifying 8 out of 10 orders. The minimum detectable frequency was 16.25 Hz, with a detection rate of 4 out of 10. The frequency that had the greatest number of false positives was 12.25 Hz. In the second testing session, the frequency that yielded the highest detection rate was 15.25 Hz, with 11 out of 12 correct orders. The lowest discernible frequency in the initial experiment was 16.25 Hz. Similarly, the frequency with the greatest number of false positives identified was 12.25 Hz.

#### 3.3.2. Online Tests on Several Subjects

The most relevant attributes of the users and the results obtained are shown in Table 7. The data were sorted in ascending order according to the accuracy and ITR obtained in each case. We also include the results of the last online test of the main user. The significance of the experiment is apparent in the variables of experience, length, and density of hair. Participant S04, characterized by short and low-density hair, had a 62% accuracy rate despite lacking prior expertise. Participant S05, characterized by an extensive and voluminous hair length, exhibited the lowest level of accuracy throughout the investigation. Subject S02, although possessing long hair, achieved a higher level of accuracy in comparison to the other subjects due to receiving a limited amount of training.

#### 3.3.3. User Survey Results

The inquiry approach was employed to assess the level of comfort and ease of use and to identify areas for improvement. Following a demonstration and comprehensive explanation of the fundamental functioning of the BCI to the participants who volunteered for the online assessments, they were subsequently asked to complete a survey. Various factors were considered, including symbol consistency, graphic representation, minimalist design, ease of learning, and error recovery, among other factors.

The findings acquired by the 5 users, across a range of qualifications consisting of 4 levels, are presented in Table 8. The percentage results were displayed as a value lower than one when taking into account a quality factor for each level. In this context, the value assigned to “low” is 0, “regular” is 0.33, “acceptable” is 0.66, and “high” is 1.0. Subsequently, it becomes possible to derive a proportional weight for each factor. The average aggregate weighting of the factors assessed in the usability tests is 83.07%, indicating a moderate to high level of importance. Furthermore, the participants were asked about the necessity of a user manual or technical support in comprehending the overall functioning of the system. It was found that 80% of the respondents deemed it essential, while the remaining 20% did not.

### 3.4. Functional Tests of the Robotic Arm

To assess the functionality of the robotic arm, individual tests were conducted on its various components. The examination encompassed the circuit’s connectivity, the initiation of the motors, and the subsequent motion, among other factors. Subsequently, recommended tests were conducted by the ISO 9283 standard for industrial manipulators [31].

The frontal plane was selected due to its requirement for the highest level of motor and geometric effort. The movements were performed inside the central plane and at the highest attainable speed, as stipulated by the established norm. The procedure entailed the demarcation of five distinct sites employing 0° and 90° lines to construct a square measuring 20 cm in length and height. Table 9 displays the outcomes acquired from a total of 30 measurements, with a focus on accuracy, precision, and repeatability. Table 10 provides a full description of the features of stabilization time, positioning time, and total movement.

### 3.5. Experimental Tests of the Assistance Device

**Figure 19:**
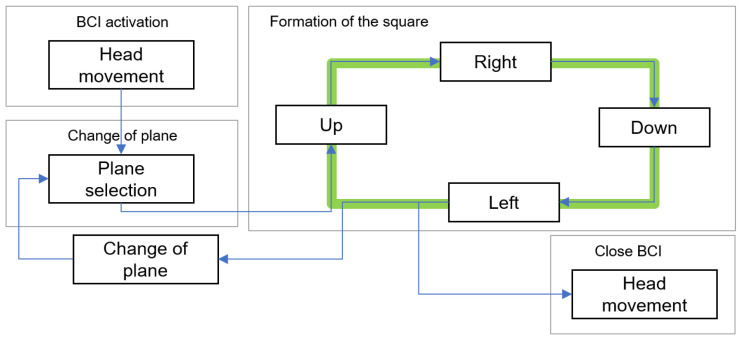
General test script for the specific task of forming a square.

The user was tasked with consecutively forming a square in each plane, as depicted in Figure 19, due to the programmed movement of the robotic arm. The objective of the study was to assess two fundamental components of the system: its level of flexibility and its capacity to effectively recover from faults.

Throughout the assignment, user S00 maintained a seated position, displaying a state of relaxation and focus, while attentively monitoring the functioning of the robotic arm and the interface. It is pertinent to note that the user in question, who participated in both the offline and online assessments of the BCI, possessed prior familiarity with the use of this technological system. It has been noted that to construct a square and alter the plane, a total of six accurate commands are necessary. In an optimal scenario, the task can be accomplished by executing a total of 18 commands.

During the initial phases of experimentation, it was observed that the presence of excessive noise and the utilization of laboratory apparatus, such as the laser cutting machine, had an adverse effect on the operational efficiency of the support device. The observed phenomenon can be attributed to the presence of interference and the distraction experienced by the user. To ensure the ideal conditions for conducting testing and obtaining reliable findings that accurately reflect the functioning of the support system, procedures were implemented to minimize the impact of detrimental external factors. The aforementioned activities encompassed the scheduling of tests, the utilization of anti-static bracelets, and the regulation of lighting conditions, among several other things.

**Figure 20:**
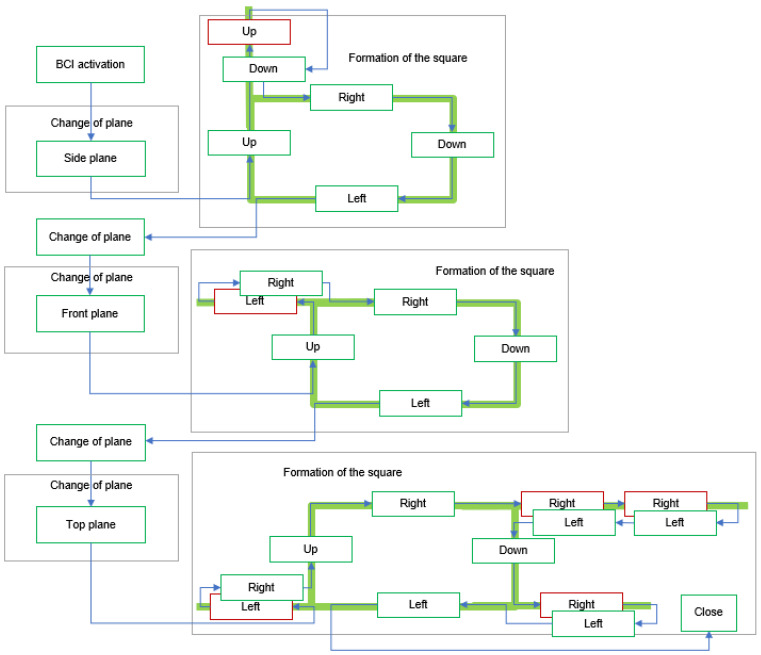
Sequence obtained in the first test session by forming a square in each plane.

At the initial session, the trajectories depicted in Figure 20 were created, forming a frame in the lateral, frontal, and upper planes. Errors were present in all three instances. Nevertheless, these errors could be rectified by employing a supplementary command. The summary results were obtained and are presented in Table 11. A total of 29 orders were necessary, out of which 23 were accurately executed. The obtained results demonstrate a mean accuracy of 79.31% for the BCI throughout the execution of the designated task.

**Figure 21:**
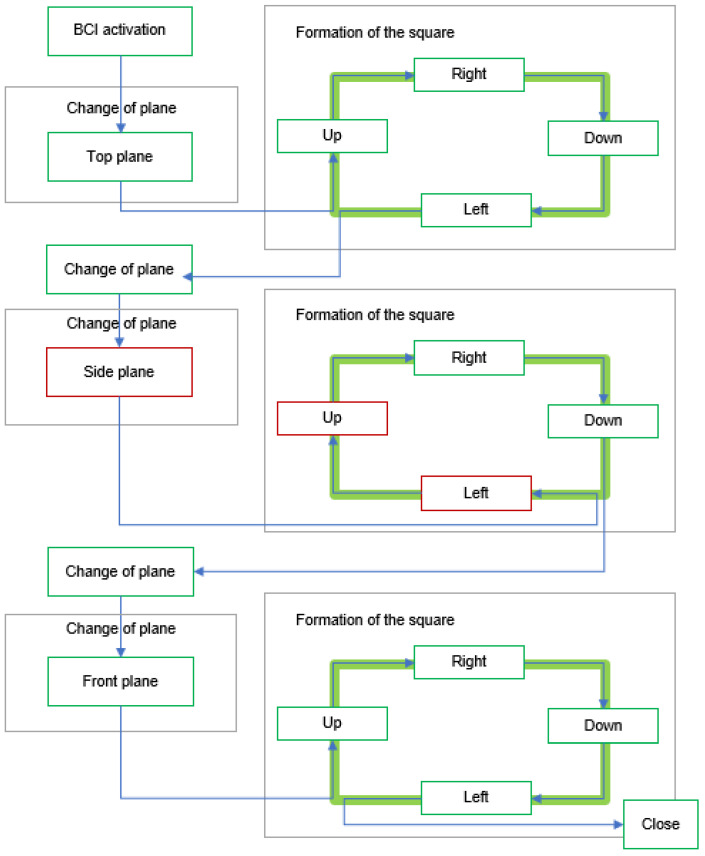
Sequence obtained in the second test session by forming a square in each plane.

In the subsequent session, three optimal trajectories were acquired, as depicted in Figure 21. Nevertheless, these findings originated as a result of three consecutive blunders that facilitated the attainment of the task of constructing the squares. The objective was successfully achieved despite deviating from the initially planned order. As a result, the system achieved an accuracy rate of 83%, with a mere three instances of wrong orders being recorded. The data shown in Table 12 demonstrate that a total of 18 instructions were employed, corresponding to an ideal task.

The performance of the integrated system can be determined by dividing the ideal number of commands by the actual number of attempts required and then multiplying the quotient by 100. Table 13 reveals that the initial session yielded a performance of 62.07%, whereas the subsequent session achieved a perfect score of 100%. The average was 81.03%.

**Figure 22:**
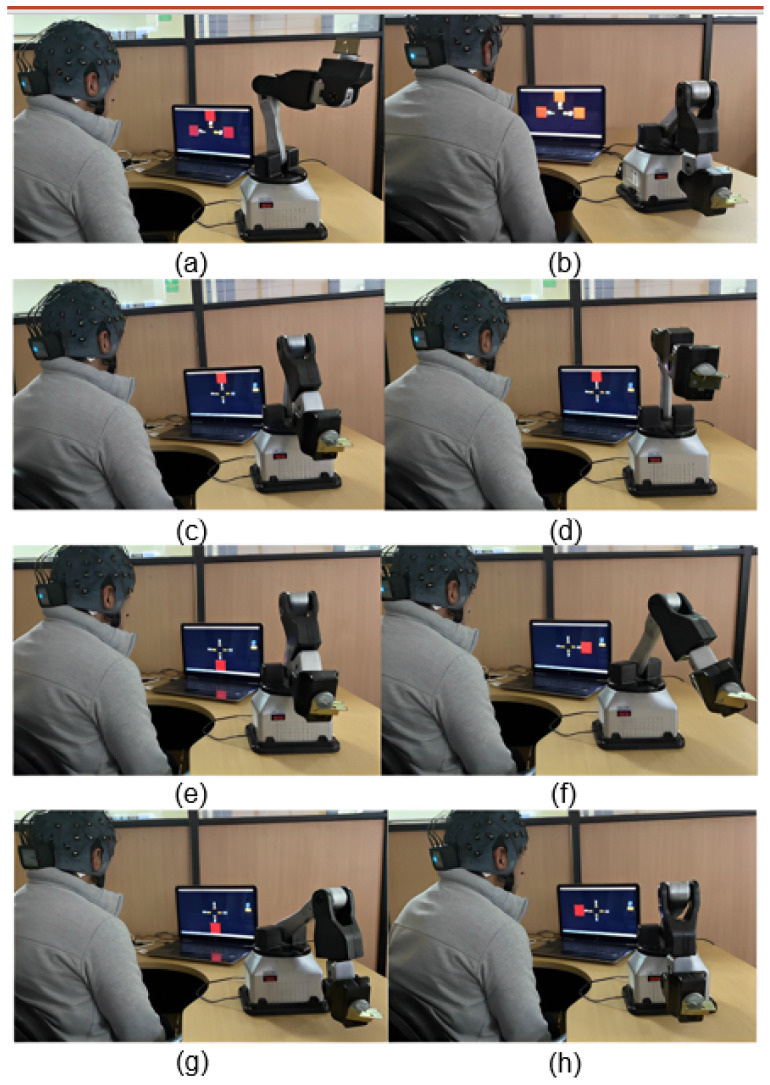
Sequence obtained in the second test session for the instructions recognized by the assistance device: (**a**) Initial location, (**b**) Side plane command, (**c**) “Up” command, (**d**) Erroneous movement, (**e**) “Down” command, (**f**) “Right” command, (**g**) “Down” command, and (**h**) “Left” command.

A graphic depiction of the support system’s actions during the first test session, forming the square in the side plane, is shown in Figure 22. The robotic arm’s initial location is displayed in Figure 22a. In the subsequent stage, denoted as Figure 22b, a proper choice is executed by transitioning to the intended side plane. The correct execution of the “Up” command in Figure 22c results in the proper movement of the robotic arm.

However, an erroneous movement is observed in Figure 22d as a result of false positive detection of the “Up” instruction. To rectify this deviation, the “Down” command is employed, as depicted in Figure 22e, enabling the user to revert to the previous location. Following this, the “Right” command in Figure 22f is accurately identified and the robotic arm executes it correctly. Next, in Figure 22g, the “Down” command is executed, finalizing the creation of the hypothetical square on the designated plane.

In Figure 22h, we can observe the correct detection of the command “Left”. Then, we continue with the instruction “Change plane”, which denotes the transition to the next plane to continue with the construction of the square. This visual study elucidates the sequential steps and necessary adjustments involved in executing a complex operation, specifically the construction of a square, with the aid of a robotic assistance system. The robotic arm successfully constructed the square without mistakes in certain instances; nevertheless, this particular example was provided to illustrate the procedure required to compensate for an erroneous command.

## 4. Discussion

A series of experimental tests were conducted to validate the functionality of the assistive device. The experimental design enabled the identification of the most effective set of algorithms, optimizing the accuracy and ITR of the BCI. Although previous studies have utilized PSD, FFT, and CCA algorithms for feature selection, as cited in references [1,3,4,7,10,24,25,26,27,30], an experimental design enables the selection of the most suitable method, taking into account relevant factors specific to the experiment, such as the type of EEG helmet, type of electrodes, and number of channels. Moreover, it is observed that the utilization of convolutional neural networks is highly frequent. In [3,5,7,22,26,29], linear discriminant analysis, quadratic discriminant analysis, and other methods are employed. PCA and ICA algorithms were evaluated during the feature extraction stage. This approach effectively reduces signal artifacts (blinks, muscle, and eye movements, among others) to simplify the feature classification stage. The literature studied solely references the usage of such algorithms in [19,21,22].

The performance of BCI was confirmed through offline tests, which demonstrated an accuracy of 83% and an ITR of 19.86 bits/min. Similarly, online tests yielded an accuracy of 75% and an ITR of 15.16 bits/min. These results are advantageous and somewhat superior to systems that integrate the design of the assistive tool, as indicated in references [1,10]. It is important to note that research does exist with accuracy levels of 90% or better, as demonstrated by references [15,17,28], and others. However, it is crucial to recognize that the use of complex algorithms directly affects the computational cost. Therefore, the objective of this research was to minimize it.

The experimental online tests conducted with a sample size of five users revealed many elements that influence the performance and efficiency of BCI in a dynamic environment. The study emphasized the notable impact of prior experience, as well as the length and density of the users’ hair. The latter is to be expected because of the variation of the contact between the EEG helmet electrodes and the scalp. The results obtained highlight that assistive technology requires customized solutions that depend on the characteristics of the users. Prior studies have indicated that it is typical to consider users’ prior training. For instance, in [4], the training duration was 10 min, whereas in [1], it spanned many weeks. This extended training period contributes to the higher accuracies achieved.

Assessing user satisfaction with the use of an assistive device is essential for identifying areas that need enhancement. Of the literature reviewed, the research in [1] mentions a simple verbal survey on robotic glove compatibility, comfort, and safety, and the study [15] specifically examines issues such as fatigue, eye strain, and visual discomfort through surveys. Users validated that the interface design has correctly rendered text and graphics, and simple and aesthetic design, among other factors. Nevertheless, areas for enhancement were discovered. These domains encompass symbol consistency and the consideration of user satisfaction concerning aspects such as color, frequency, and duration of stimuli. Validation of these features, which are important for enhancing the user experience, needs an experimental design that meticulously takes into account these factors. Implementing modifications in response to this validation process has the potential to enhance or reduce the tool’s usefulness and adaptability in practical scenarios.

The 6-DOF robotic arm has been purposefully designed and built to cater to the requirements of individuals with disabilities. This sets it apart from commercial robotic arms, which are primarily designed for industrial purposes, as evidenced by research [5,6]. Although the system was able to recover positions after incorrect commands, it was observed that the number of commands required increased, specifically two additional attempts for each incorrect choice. Average accuracy reached 79% and 83% in two consecutive sessions, demonstrating consistency in performance. Increased user experience or changes in the experimental environment, such as reduced light reflection on the computer screen, may have contributed to the slight improvement in accuracy compared to the online tests. This analysis underlines the system’s capability for specific tasks and highlights its adaptability in improved environments. Adopting the ISO 9283 standard [31] for evaluating the robotic arm’s performance establishes uniform parameters, ensuring a precise and unbiased evaluation. The robotic arm’s results, which have an accuracy of ±0.85 cm and a repeatability of ±1.49 cm, demonstrate its good performance.

This assistance device possesses notable benefits that distinguish it within the realm of support. Integrating online task-specific user testing serves to evaluate the tool in a better way and reveals aspects that impact its performance. Targeted adjustments and enhancements can be made as a result. The incorporation of a 6-DOF robotic arm, designed for the user, enhances the range of possible uses for the tool in assistive settings. Additionally, it decreases the tool’s expense by utilizing 3D printing technology and low-cost electronic gadgets.

Although the developed support tool has clear benefits, it is important to acknowledge its limitations as well. The presentation of rapidly changing visual stimuli can lead to visual fatigue, suggesting the need for strategies to mitigate this effect in the long term. Furthermore, the extended use of the EEG helmet may cause discomfort as a result of continuous contact with the scalp. This emphasizes the significance of investigating ergonomic design alternatives and utilizing lighter materials to enhance user comfort. Utilizing 3D printing techniques decreases the expense of the robotic arm, but the cost of the EEG helmet still plays a substantial role in the total expense of the device, raising concerns about affordability. Subsequently, employing low-cost EEG headsets could be regarded as a viable option to alleviate this cost issue. Furthermore, there is noticeable diversity in user adaptation, with certain individuals demonstrating excellent aptitude for concentration and rapid adjustment, while others are uncomfortable and have difficulty getting used to it. Investigating customized training and adaptation tactics may enhance the entire user experience.

## 5. Conclusions

This study emphasizes the potential of combining a BCI with a 6-DOF robotic arm as a valuable tool for assistance purposes. The experimental design, which focused on feature extraction and selection, determined that PCA and CCA algorithms were the most effective in their respective stages compared to ICA, PSD, and FFT. The testing revealed various aspects that either hindered or enhanced the functioning of the BCI. These factors include the length and density of the user’s hair, familiarity with SSVEP stimuli, and degree of focus. The outcomes are noteworthy, exhibiting accuracies of 75% during the online session and 83% during the offline session, employing five control frequencies (12.25, 13.25, 14, 15.25, and 16.25 Hz). Surveys verified that the interface is both intuitive and user-friendly. The algorithms reduced the computational cost as they utilize mathematical correlations instead of complex neural networks. This enables the tool to be used online without the need for a machine with high processing capabilities.

The assistive device, constructed to meet the user’s specifications, guarantees important attributes such as slow velocities, steady motions with little vibration, and an effective operational area. The robotic arm possesses a high level of stability in its movements, with an accuracy of ±0.85 cm and a repeatability of ±1.49 cm. This enables precise manipulation of the location and orientation of objects in three-dimensional space. The experimental testing indicated a high level of functionality by successfully creating a hypothetical square in each demonstration, with accuracies of 79% and 83%. In addition, the movement facilitated by the robotic arm allows the user to learn about and interact with this technology and then adapt it to more specific assistive activities.

Several potential areas for future research arise in the continual pursuit to enhance and broaden the scope of this study. A potential future trajectory is merging the robotic arm with a wheelchair, thereby optimizing support for individuals with severe disabilities. By employing computer vision, it is possible to program precise trajectories for performing common actions like feeding and cleaning teeth. This would enhance the user’s autonomy and self-reliance. Integrating eye-tracking technologies has the potential to enhance detection capabilities, hence improving the overall efficiency of the system. Likewise, the development of a customized EEG helmet that precisely positions electrodes on targeted areas of the brain or the utilization of affordable EEG headsets can improve the user’s comfort level. Integrating virtual reality components is proposed as a tactic to mitigate visual exhaustion and enhance the user’s overall experience. Investigating new paradigms for BCI or decreasing stimulus duration are further avenues of research that could enhance the effectiveness and real-world implementation of this assistive tool. These future work ideas indicate promising prospects for further research that will enhance the ongoing development of this assistive technology.

## Figures and Tables

**Table 1:** Comparison of similar research conducted.

Authors	Assistance Tool	Control and Operation Form	BCI Procedures	Results Evaluated
Guo, N. et al. [1]	Soft robotic glove for rehabilitation.	BCI-SSVEP. Online tests with 2 frequencies and 3 s.	Emotiv EPOC. Algorithms: CCA.	Users: 10 Accuracy: 65.9%
Meijneke, C. et al. [2]	Symbitron exoskeleton. DOF: 8	Neuromuscular controller.	BCI is not used.	Speed and stride length recording. Users: 3
Chu, Y. et al. [3]	Robot-assisted rehabilitation system. DOF: 3	BCI-SSVEP. Online tests with 3 frequences and 7 s.	Emotiv EPOC. Algorithms: PSD, LDA.	Speed variation. Users: 6 Accuracy:75.%
Siribunyaphat, N. et al. [4]	Simulated wheelchair	BCI-SSVEP. Online tests with 4 frequences and 4 s.	Emotiv EPOC. Algorithms: PSD	Routes. Users: 12 Accuracy: 85%
Quiles, E. et al. [5]	Industrial robotic arm. DOF: 6	BCI-SSVEP. Online tests with 3 frequences and 7 s.	Enobio. Algorithms: PSD, LDA.	Robot movement. Users: 5 Accuracy: 72%
Chen, X. et al. [6]	Industrial robotic arm. DOF: 6	BCI-SSVEP. Online tests with 15 frequences and 4 s.	Neuracle Algorithms: FBCCA.	Robot movement. Users: 12 Accuracy: 92%
Ravi, A. et al. [7]	No	BCI-SSVEP and SSMVEP. Online tests with 4 frequencies and 2 s.	g.USBamp. Algorithms: CCA, C-CNN.	Users: 26 Accuracy: 83%.
Yuan, Z. et al. [10]	Pedaling training system.	BCI: Patient’s attention index. Online tests.	Energy ratio of band signals. Single-channel.	Users: 30 Lower limb motor function increased.
Liu, B. et al. [15]	Speller.	BCI-SSVEP. Online tests with 40 frequences and 1 s.	Neuracle. Algorithms: CCA, TRCA, TDCA.	Users: 12 Accuracy: 97%.
Kancaoglu, M. et al. [16]	No	BCI-SSVEP. Online tests with 5 frequences and 1 s.	Neuracle. Algorithms: CCA, PSD, LASSO.	Users: 12 Accuracy: 50%.
Mannan, M. et al. [17]	Speller.	BCI-SSVEP. Online tests with 6 frequences and 1 s.	gUSBAmp. Eyelink 1000. Algorithms: CCA.	Write a sentence. Users: 12 Accuracy: 89%.
Latreche, A. et al. [18]	Home telerehabili- tation platform.	Website for telerehabilitation and track.	BCI is not used. Use of artificial intelligence.	Users: 2 The patients have better movement in some weeks.
The authors in this work	Robot arm designed. DOF: 6	BCI-SSVEP. Online tests with 5 frequences and 4 s.	g Nautilus Research. Algorithms: PCA, ICA, CCA, FFT, PSD.	Imaginary square. Users: 6 Accuracy: 75%.

**Table 2:** Characteristics of preprocessing filters.

Type	Characteristics
Butterworth Bandpass	5–30 Hz
Notch	60 Hz
CAR	
Additional filter	Noise Reduction

**Table 3:** Experimental design.

Factors	Level 1	Level 2	Level 3
Extraction	PCA	ICA	-
Selection	CCA	FFT	PSD

**Table 4:** Results of the experimental design.

Extraction	Selection	Mean Accuracy	Mean ITR (bits/min)
PCA	CCA	0.83	20.45
PCA	FFT	0.69	13.42
PCA	PSD	0.67	11.33
ICA	CCA	0.61	8.58
ICA	FFT	0.49	4.84
ICA	PSD	0.47	4.32

**Table 5:** Offline test results.

Parameters	Value
Accuracy (%)	83
ITR (bits/min)	19.86

**Table 6:** Results of the online tests for the main user in two sessions.

Parameters	Session 1	Session 2
Accuracy (%)	64	75
ITR (bits/min)	9.89	15.16

**Table 7:** Online test results and user characteristics.

Subject	S05	S03	S01	S02	S04	S00
Age	24	24	24	24	24	23
Sex	Female	Male	Male	Female	Male	Male
Hair density	High	Medium	Medium	Low	Low	Low
Hair length	Long	Medium	Medium	Long	Very short	Short
Experience	No	No	No	Relative	No	Yes
Accuracy (%)	18	26	28	46	62	75
ITR (bits/min)	0.028	0.228	0.397	3.698	9.058	15.160

**Table 8:** User survey results of the BCI.

Factors	L 0	R 0.33	A 0.66	H 1.00	Average (%)
Symbol consistency			0.80	0.20	72.80
Correctly rendered text and graphics			0.20	0.80	93.20
Simple and aesthetic design		0.20	0.20	0.60	79.80
Easy-to-learn interface management			0.60	0.40	79.60
Comfort of color, frequency, and time of stimulation		0.20	0.40	0.40	73.00
It is intuitive to recover an action after making a mistake				1.00	100.00
Average					83.07

Note. L low, R regular, A acceptable, and H high.

**Table 9:** Robotic arm test results using ISO 9283 Standard [31].

Parameters	Position (cm)	Orientation *X* Axis (°)	Orientation *Y* Axis (°)	Orientation *Z* Axis (°)
Position accuracy	±0.85	±0.75	±1.14	±0.57
Trajectory accuracy	±1.0	±1.15	±1.35	±0.82
Position precision	0.5	0.43	0.52	0.29
Position repeatability	0.85 ± 1.49	±1.28	±1.55	±0.88
Trajectory precision	0.57	0.23	0.64	0.28
Trajectory repeatability	1 ± 1.7	±0.68	±1.92	±0.85

**Table 10:** Robotic arm time test results using ISO 9283 Standard [31].

Time Parameters	Time (s)
Stabilization time	0.7
Minimum time of positioning	1.0
Total movement time	1.7

**Table 11:** Results of the session I-General tests.

Plane	Required Commands	Correct Commands	Accuracy (%)
Side	8	7	87.50
Front	8	7	87.50
Top	13	9	69.23
Total	29	23	79.31

**Table 12:** Results of the session II-General tests.

Plane	Required Commands	Correct Commands	Accuracy (%)
Side	6	6	100.00
Front	6	3	50.00
Top	6	6	100.00
Total	18	15	83.33

**Table 13:** Performance results of the assistive device.

Session	Ideal Required Commands	Required Commands	Performance (%)
1	18	29	62.07
2	18	18	100.00
Average			81.03

## Data Availability

Data are contained within the article.

## List of Abbreviations

BCI	Brain–computer interface
SSVEP	Visual-evoked potential paradigm
DOF	Degrees of freedom
EEG	Electroencephalography
ITR	Information transfer rate
PCA	Principal Component Analysis
ICA	Independent Component Analysis
FFT	Fast Fourier Transform
PSD	Power Spectral Density
CCA	Canonical Correlation Analysis
CAR	Common Average Reference
